# Supplementary material for: Developmental exposure to non-dioxin-like polychlorinated biphenyls promotes sensory deficits and disrupts dopaminergic and GABAergic signaling in zebrafish
Source: Commun Biol. 2021 Sep 24;4:1129. doi: 10.1038/s42003-021-02626-9 (PMC8463681; doi:10.1038/s42003-021-02626-9)
Supplement: Supplementary file 1 — Supplementary Information [file 42003_2021_2626_MOESM1_ESM.pdf]

Supplementary Information

Developmental exposure to non-dioxin-like polychlorinated biphenyls promotes sensory deficits and disrupts dopaminergic and GABAergic signaling in zebrafish

Nadja R. Brun, Jennifer M. Panlilio, Kun Zhang, Yanbin Zhao, Evgeny Ivashkin, John J. Stegeman, Jared V. Goldstone

Supplementary Table 1. Replicate number per treatment group used in the startle response assay and statistical results. The *p*-values below 0.05 are bold.

**Treatment**	***n***	**In comparison to**	***n***	***p*-value for Fraction responding**				***p*-value for SLC bias**			
				32 dB	38 dB	41 dB	42 dB	32 dB	38 dB	41dB	42 dB
DMSO	479 [a]	1000 nM PCB153 [b]	139	**<.0001**	**<.0001**	**.0003**	**.0003**	**<.0001**	**<.0001**	**<.0001**	**<.0001**
		100 nM PCB153 [b]	234	**<.0001**	**<.0001**	**<.0001**	**<.0001**	**<.0001**	**<.0001**	**<.0001**	**<.0001**
		30 nM PCB153 [b]	147	**.0037**	**.0008**	**.0002**	**.0002**	**<.0001**	**.0002**	**<.0001**	**<.0001**
		10 nM PCB153 [b]	103	.59	.603	.787	.147	**.0028**	**.0038**	**.0035**	**.0025**
		1000 nM PCB138 [b]	110	**.0001**	**.0001**	**.0001**	**.0001**	.2289	**.0003**	**<.0001**	**<.0001**
		1000 nM PCB118 [b]	100	.0862	**<.0001**	.223	.025	**<.0001**	**<.0001**	**.0002**	**.0005**
		1000 nM PCB52 [b]	105	**.0009**	**<.0001**	**<.0001**	**<.0001**	.4902	**.0024**	**.0008**	**<.0001**
		1 nM PCB126 [b]	83	**.0014**	**.0008**	.751	.088	**.0064**	**<.0001**	**<.0001**	**<.0001**
		DMSO + L-DOPA 4-120 hpf [b]	62	.247	.182	.203	.756	.8741	**.0243**	**.0100**	**.0085**
		DMSO + Quinpirole 4-120 hpf [b]	125	.562	.827	.61	.395	.3302	.1584	.877	.289
DMSO 24-120 hpf	80	PCB153 24-120 hpf	83	**.0006**	**<.0001**	**.0071**	**<.0001**	.248	**.0017**	**<.0001**	**<.0001**
PCB153 4-120 hpf	62	PCB153 + L-DOPA 4-120 hpf	139	**.0062**	**<.0001**	**<.0001**	**<.0001**	.349	.0054	.212	.2710
DMSO 0 min	158	DMSO 30 min	172	.1123	.113	.543	.518	**.0243**	**.0283**	.159	.9840
PCB153 0 min	58	PCB153 30 min	58	.962	.763	.478	**.0343**	.7228	.1137	.8943	.9600
DMSO before 5-HT	49	after 30 min 5-HT	49	**.0009**	**<.0001**	**<.0001**	**<.0001**	.329	.155	.986	>.9999
DMSO before 5-HTP	79	after 30 min 5-HTP	80	.2935	.834	.392	.755	**.0004**	**.0019**	**<.0001**	**<.0001**
DMSO before L-DOPA	93	after 30 min L-DOPA	94	.0612	.221	.373	.244	.123	**.0001**	**<.0001**	**.0009**
DMSO before L-tyrosine	85	after 30 min L-tyrosine	85	.635	.539	.32	.64	.239	.155	**.0073**	.1707
DMSO before Quinpirole	101	after 30 min Quinpirole	101	**.0001**	**<.0001**	.169	**.0202**	.1218	.671	**.0006**	**.0016**
DMSO before Haloperidol	178	after 30 min Haloperidol	158	.617	.457	.1037	.3693	**<.0001**	**<.0001**	**<.0001**	**.0015**
PCB153 before Haloperidol	81	after 30 min Haloperidol	73	.952	.5070	**.0002**	.978	**.0003**	**<.0001**	**<.0001**	**.0002**
DMSO before Bicuculline	160	after 30 min Bicuculline	159	.144	.6359	.3031	.1562	. 0629	.0913	**.0185**	**.0001**
PCB153 before Bicuculline	133	after 30 min Bicuculline	137	.8430	.914	.864	.6639	.167	.0855	.0805	.2109
DMSO before Nipecotic Acid	152	after 30 min Nipecotic Acid	152	.3171	.6568	.7205	.385	.1969	.8571	**.0040**	.260
PCB153 before Nipecotic Acid	166	after 30 min Nipecotic Acid	166	**.0222**	.445	.591	.664	.988	.574	.363	.527

[a] total *n* for DMSO
[b] in comparison to DMSO of the respective trial date

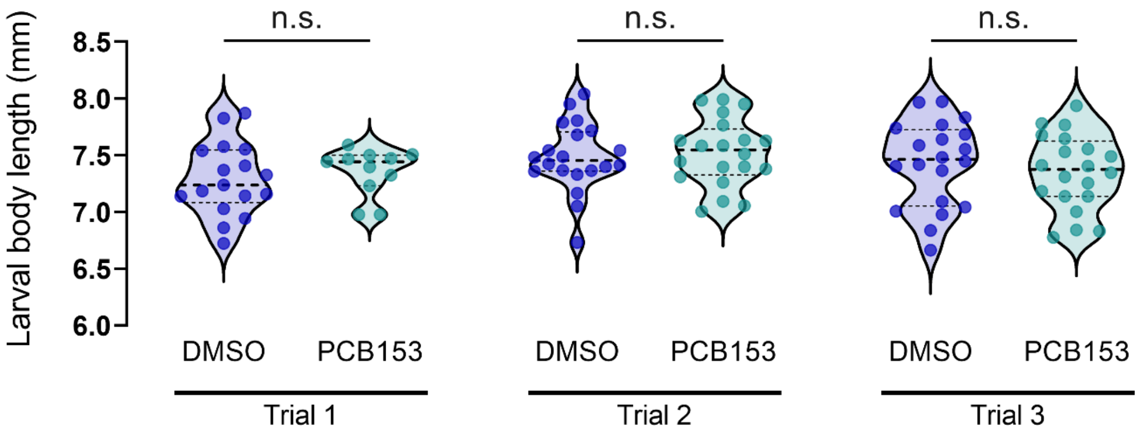

Supplementary Figure 1. Larval length. No difference (Welch's t-test) in larval length at six dpf was determined comparing solvent control with 1000 nM PCB153 treated larvae. Larval body length was measured from snout to tail-fin end. All data points are biologically independent replicates from three independent experiments (trials). Trial 1: DMSO *n* = 17, PCB153 *n* = 11, Trial 2: DMSO *n* = 20, PCB153 *n* = 20, Trial 3: DMSO *n* = 20, PCB153 *n* = 20. Dashed lines represent median and quartiles.

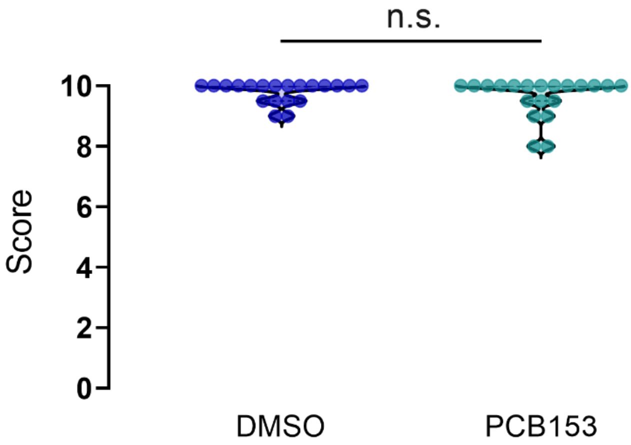

Supplementary Figure 2. Embryonic mechanosensory response. The activity of Rohon-Beard cells in the trunk zebrafish embryos was tested by a light touch at 26 hpf. Each embryo was touched ten times and individual responses were scored as follows: 0, no response; 0.5, light response, 1, coiling in the opposite direction of the touch stimulus. The total score was calculated as the sum of individual scores. Statistical analysis (Mann-Whitney test) revealed no difference between DMSO and 1000 nM PCB153 treated embryos (*n* = 20 per group). Data points represent biologically independent replicates from two independent experiments. Dashed lines represent median and quartiles.

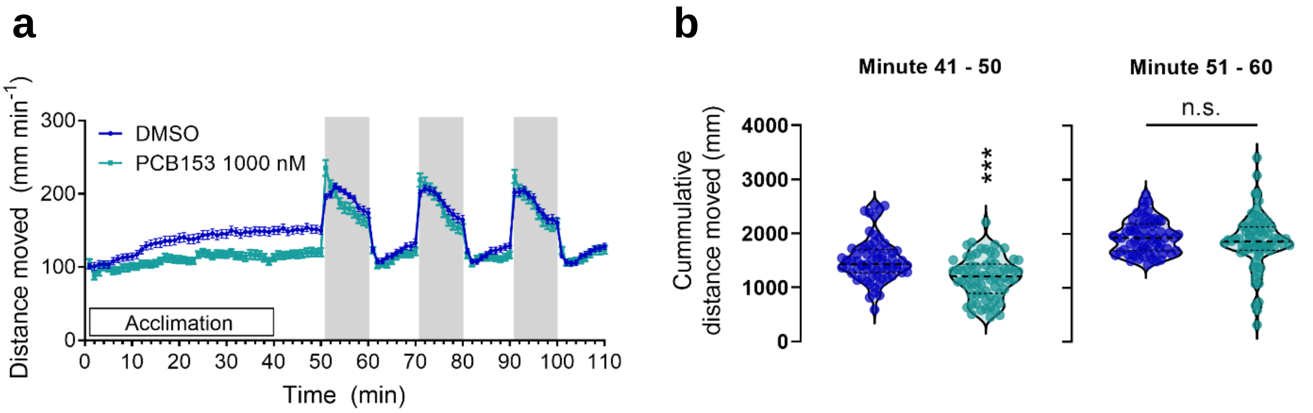

Supplementary Figure 3. Larval locomotion. (a) Larval locomotion was tracked at six dpf over 110 minutes with three dark phases of 10 minutes each. Error bars represent the mean ± SEM. **(b)** The cumulative spontaneous swimming activity (41-50 min) of PCB153 (n = 67) treated larvae is lower in comparison to the solvent control (n = 68) treated larvae, $t(132.4) = 4.854$, $p < .0001$. No difference between control and treatment is observed in the alternating light/dark phases. All data points represent biologically independent replicates from three independent experiments. Dashed lines represent median and quartiles.

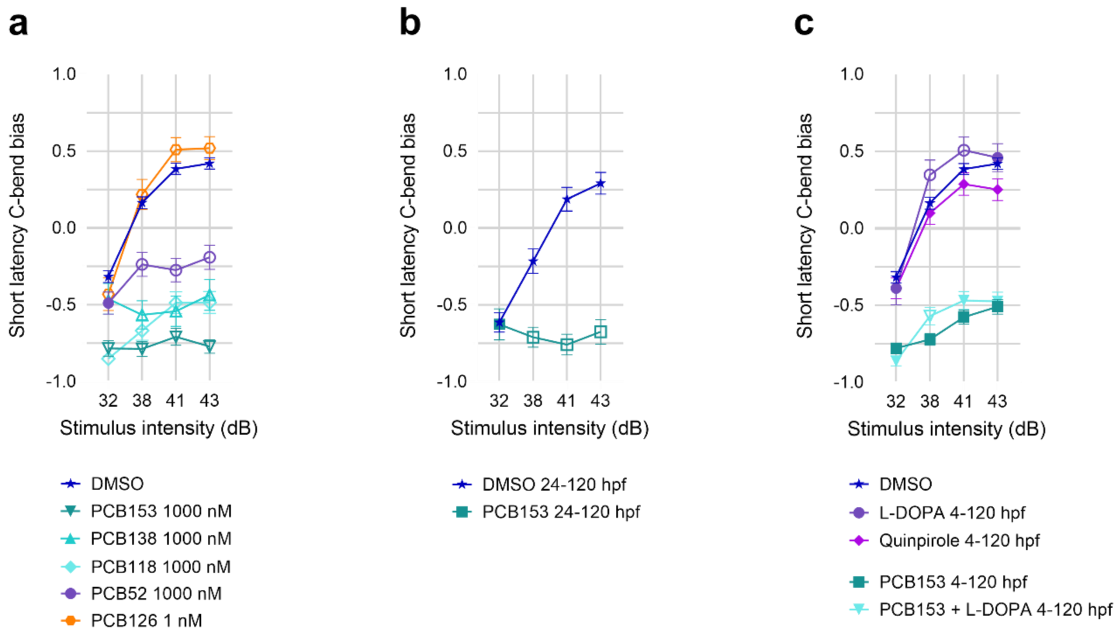

d

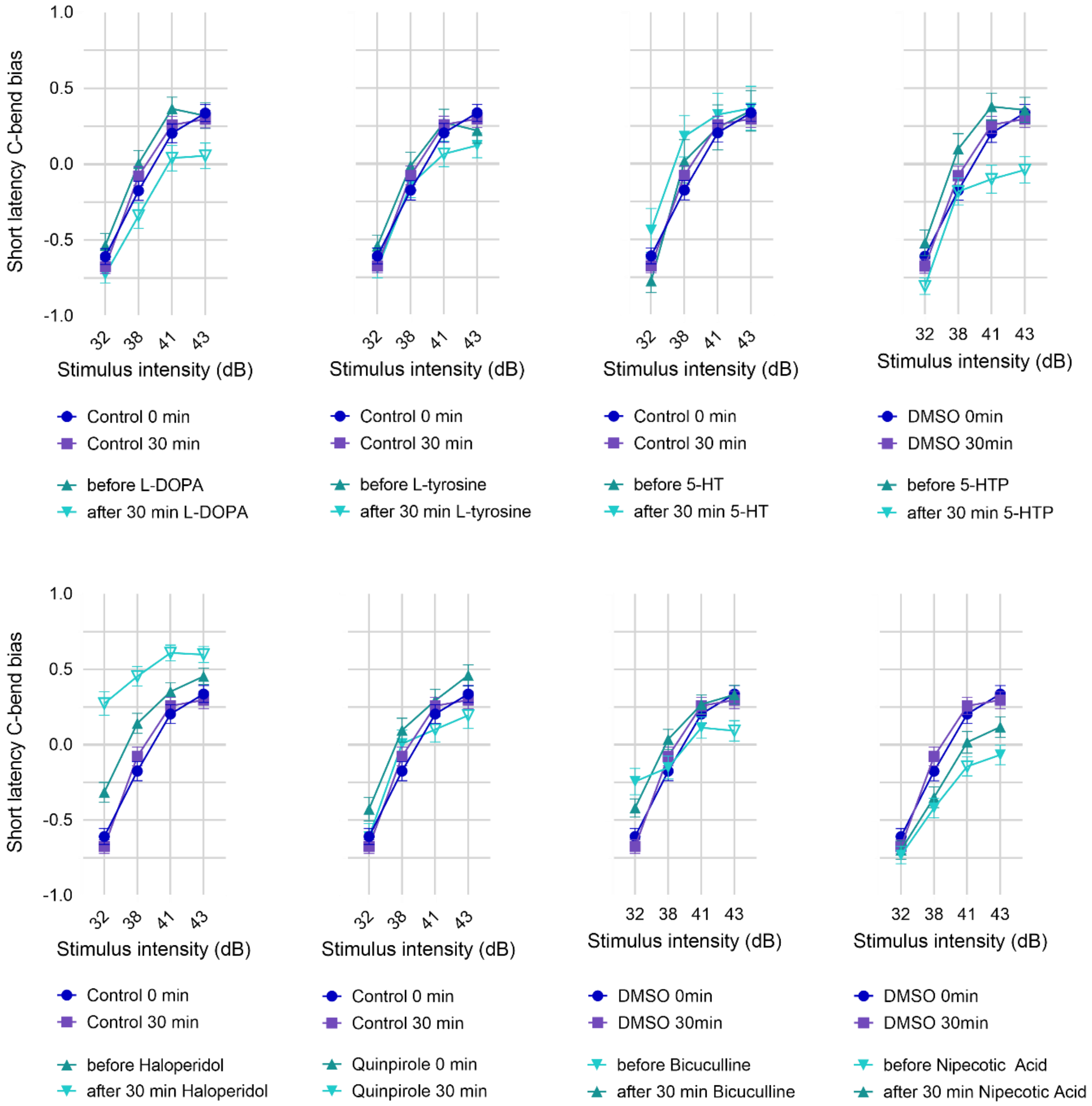
Short latency C-bend bias
1.0
0.5
0.0
-0.5
-1.0
32
38
41
43
Stimulus intensity (dB)
Control 0 min
Control 30 min
before L-DOPA
after 30 min L-DOPA
Control 0 min
Control 30 min
before L-tyrosine
after 30 min L-tyrosine
Control 0 min
Control 30 min
before 5-HT
after 30 min 5-HT
DMSO 0min
DMSO 30min
before 5-HTP
after 30 min 5-HTP
Control 0 min
Control 30 min
before Haloperidol
after 30 min Haloperidol
Control 0 min
Control 30 min
Quinpirole 0 min
Quinpirole 30 min
DMSO 0min
DMSO 30min
before Bicuculline
after 30 min Bicuculline
DMSO 0min
DMSO 30min
before Nipecotic Acid
after 30 min Nipecotic Acid

e

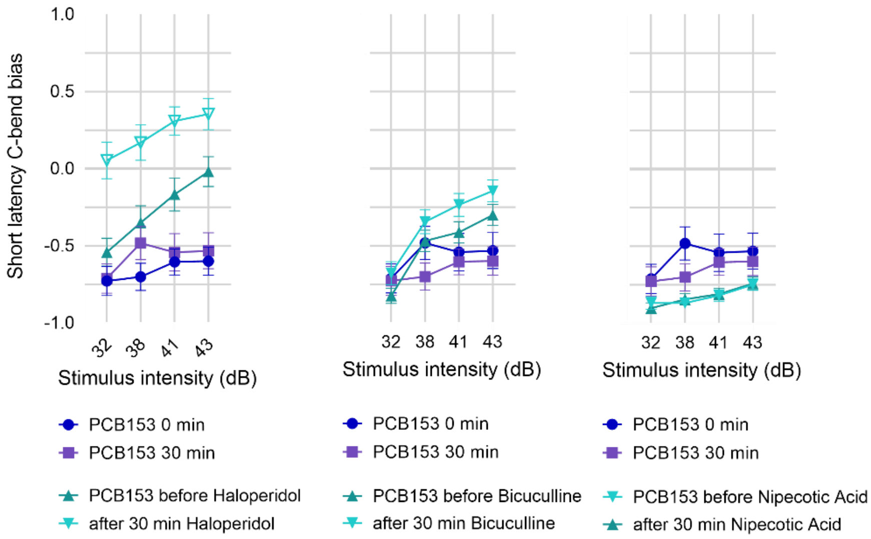
Short latency C-bend bias
1.0
0.5
0.0
-0.5
-1.0
32
38
41
43
Stimulus intensity (dB)
PCB153 0 min
PCB153 30 min
PCB153 before Haloperidol
after 30 min Haloperidol
PCB153 0 min
PCB153 30 min
PCB153 before Bicuculline
after 30 min Bicuculline
PCB153 0 min
PCB153 30 min
PCB153 before Nipecotic Acid
after 30 min Nipecotic Acid

Supplementary Figure 4. Short latency C-bend bias at different stimulus intensities. Shifts from long latency C-bend (LLC) to short latency C-bend (SLC) with increasing stimulus intensities in control groups (DMSO *n* = 479, DMSO 24-120 hpf *n* = 80, DMSO 0 and 30 min *n* =161) in comparison to (**a**) different non-dioxin-like-PCBs (PCB153 *n* = 139, PCB138 *n* = 110, PCB118 *n* = 100, PCB52 *n* = 105) and the dioxin-like PCB126 (*n* = 83), (**b**) exposure to 1000 nM PCB153 excluding gastrulation (24-120 hpf; PCB153 *n* = 83), (**c**) exposure throughout development (4-120 hpf) to L-DOPA 1 mM (*n* = 62) or Quinpirole 10 µM (*n* = 125) and PCB153 100 nM alone (*n* = 62) and with co-exposure to L-DOPA (*n* = 139), (**d**) neurotransmitter modulators (L-DOPA *n* = 90, L-tyrosine *n* = 85, 5-HT *n* = 49, 5-HTP *n* = 78, Haloperidol *n* = 177, Quinpirole *n* = 101, Bicuculline *n* = 163, Nipecotic Acid *n* = 152) for 30 min in DMSO treated larvae at 6 dpf, (**e**) neurotransmitter modulators (Haloperidol *n* = 94, Bicuculline *n* = 143, Nipecotic Acid *n* = 166) for 30 min in 100 nM PCB153 treated larvae at 6 dpf and 100 nM PCB153 only (*n* = 58). Values are presented as mean ± SEM. All data points are biologically independent replicates from at least three independent experiments. Hollow symbols indicate a significant difference to the respective controls (binomial GLM, *p* < 0.01).

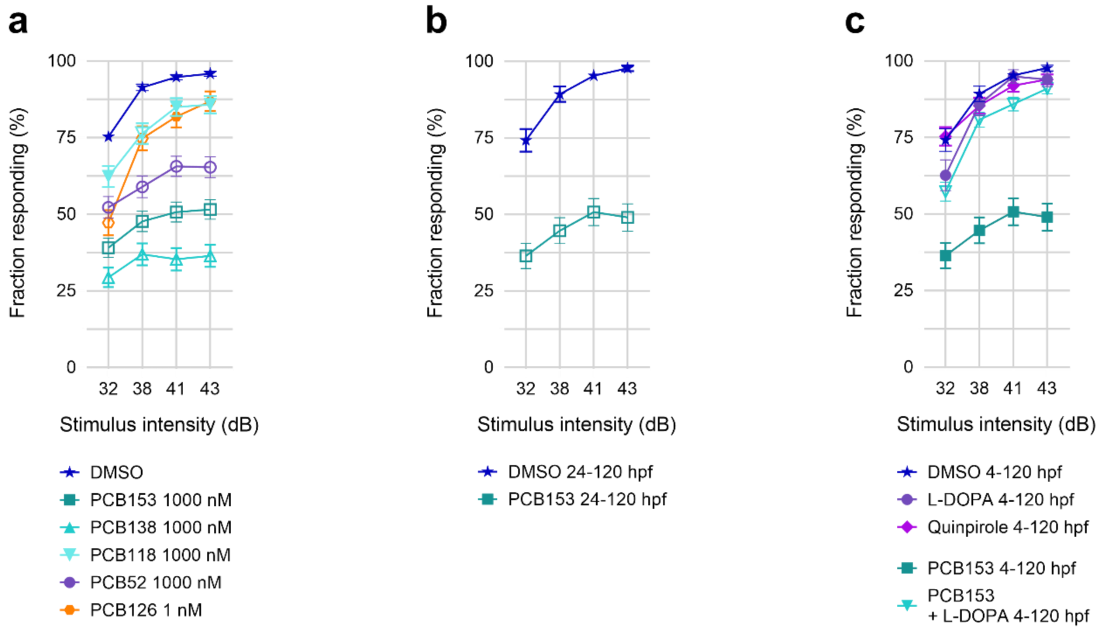

d

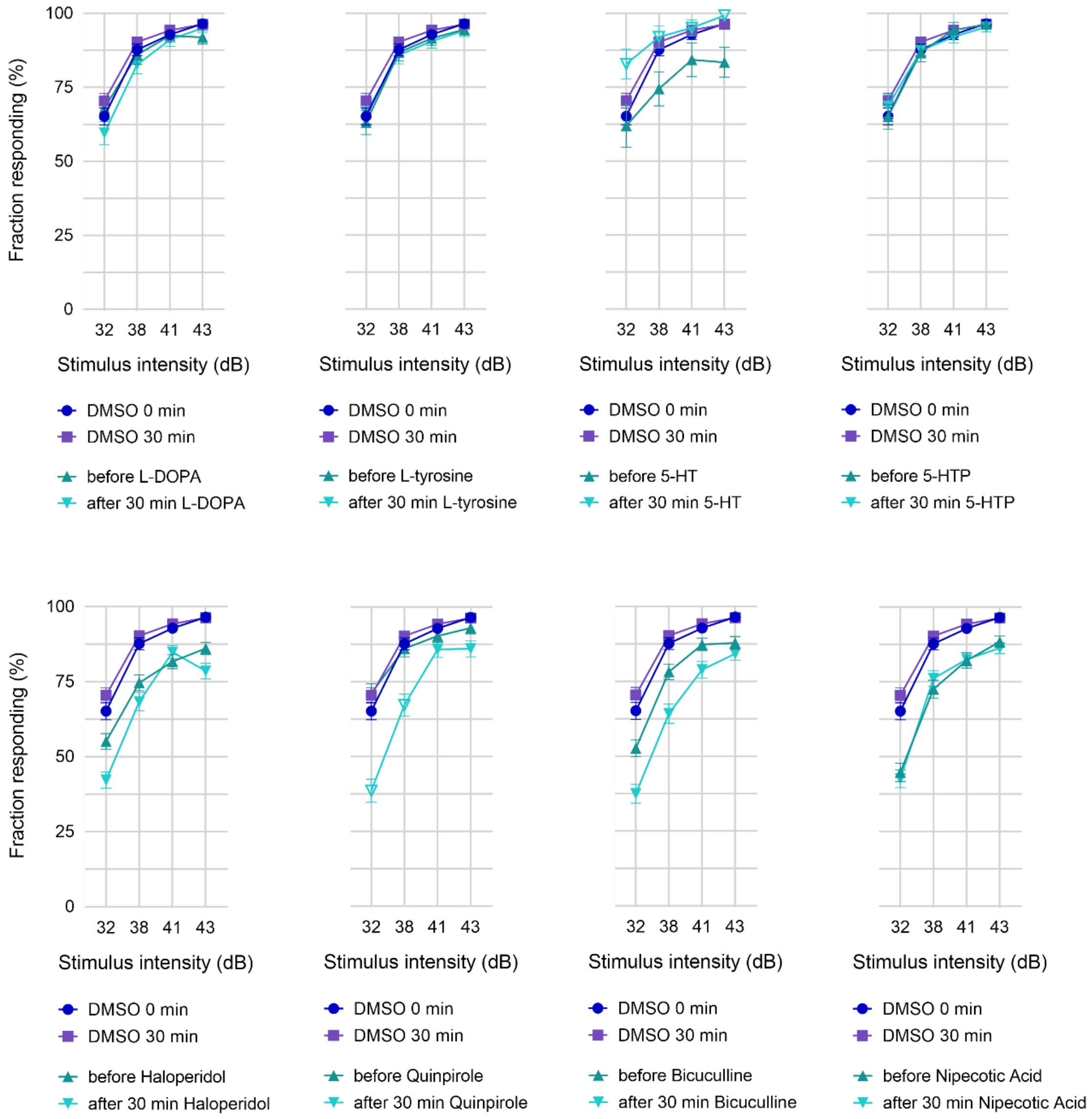
Fraction responding (%)
100
75
50
25
0
32 38 41 43
Stimulus intensity (dB)
DMSO 0 min
DMSO 30 min
before L-DOPA
after 30 min L-DOPA
before L-tyrosine
after 30 min L-tyrosine
before 5-HT
after 30 min 5-HT
before 5-HTP
after 30 min 5-HTP
before Haloperidol
after 30 min Haloperidol
before Quinpirole
after 30 min Quinpirole
before Bicuculline
after 30 min Bicuculline
before Nipecotic Acid
after 30 min Nipecotic Acid

e

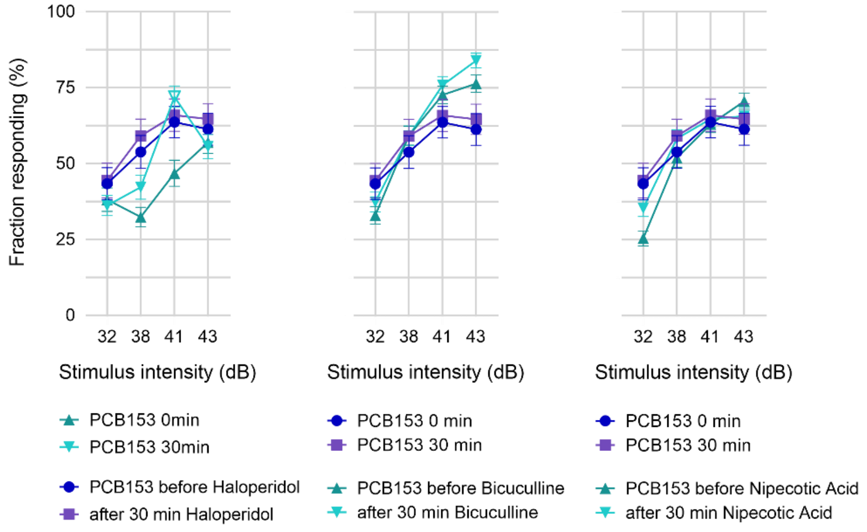
Fraction responding (%)
100
75
50
25
0
32 38 41 43
Stimulus intensity (dB)
PCB153 0min
PCB153 30min
PCB153 before Haloperidol
after 30 min Haloperidol
PCB153 0 min
PCB153 30 min
PCB153 before Bicuculline
after 30 min Bicuculline
PCB153 before Nipecotic Acid
after 30 min Nipecotic Acid

Supplementary Figure 5. Startle response rate at different stimulus intensities. Response rate in control groups (DMSO *n* = 479, DMSO 24-120 hpf *n* = 80, DMSO 0 and 30 min *n* = 161) in comparison to (**a**) different non-dioxin-like-PCBs (PCB153 *n* = 139, PCB138 *n* = 110, PCB118 *n* = 100, PCB52 *n* = 105) and the dioxin-like PCB126 (*n* = 83), (**b**) 1000 nM PCB153 excluding gastrulation (24-120 hpf; *n* = 83), (**c**) exposure throughout development (4-120 hpf) to L-DOPA 1 mM (*n* = 62) or Quinpirole 10 µM (*n* = 125) and 100 nM PCB153 alone (*n* = 62) and with co-exposure to L-DOPA (*n* = 139), (**d**) neurotransmitter modulators (L-DOPA *n* = 90, L-tyrosine *n* = 82, 5-HT *n* = 49, 5-HTP *n* = 78, Haloperidol *n* = 177, Quinpirole *n* = 101, Bicuculline *n* = 163, Nipecotic Acid *n* = 152) for 30 min in DMSO treated larvae at 6 dpf, (**e**) neurotransmitter modulators (Haloperidol *n* = 94, Bicuculline *n* = 143, Nipecotic Acid *n* = 166) for 30 min in 100 nM PCB153 treated larvae at 6 dpf and 100 nM PCB153 only (*n* = 58). Values are presented as mean ± SEM. All data points are biologically independent replicates from at least three independent experiments. Hollow symbols indicate a significant difference to the respective controls (binomial GLM, $p < 0.01$).

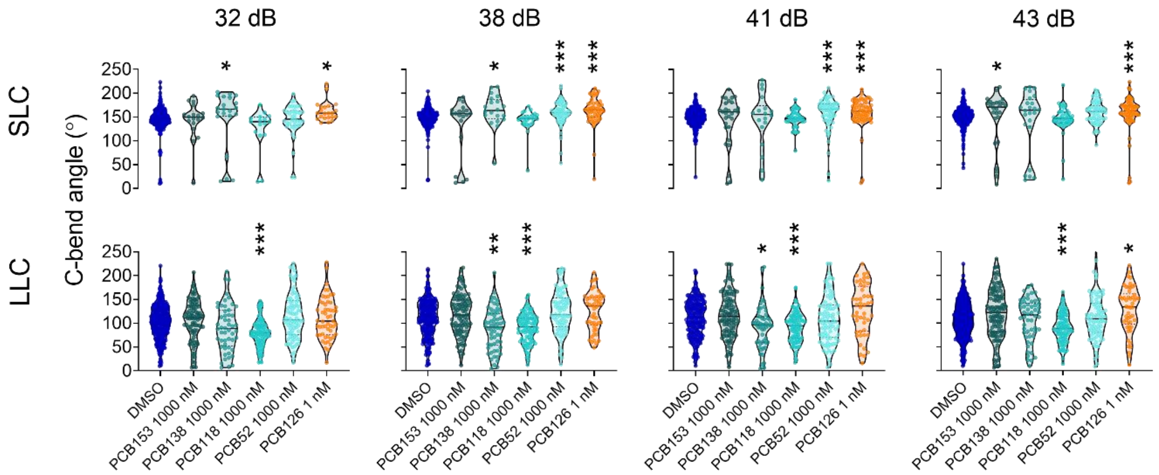

Supplementary Figure 6. Bend angle of C-start. Larval C-bend angle of control larvae remain relatively constant at different auditory stimulus intensities but vary between short latency C-bend (SLC; *n* = 356, median = 152.1˚) and long latency C-bend (LLC; *n* = 225, median = 107.9˚). PCB153 (1000 nM) exposure resulted in an increased SLC bend angle at the highest stimulus intensity for those few larvae that exhibited an SLC (43 dB, *n* = 26, *p* = .0456) while PCB52-treated larvae had increased SLC bend angles at two intermediate stimulus intensities (38 dB: *n* = 57, *p* = 0.0009 and 41 dB: *n* = 59, *p* = .0002). Across all stimulus intensities, PCB126 treated larvae show a significantly increased bend angle during SLC start (32 dB: *n* = 52, *p* < .0127; 38 dB: *n* = 68, *p* = < .0001, 41 dB: *n* = 59, *p* < .0001, 43 dB: *n* = 67, *p* = .0002) and PCB118 treated larvae exhibit a decreased bend angle during LLC start (32 dB: *n* = 92, *p* < .0001; 38 dB: *n* = 88, *p* = < .0001, 41 dB: *n* = 89, *p* < .0001, 43 dB: *n* = 87, *p* = 0.0005) in comparison to DMSO treated larvae of respective stimulus intensity. Data points represent biologically independent replicates from at least three independent experiments with black horizontal bars indicating the median. SLC was analyzed using a Kruskal-Wallis and LLC with a Welch ANOVA. Asterisks indicate significant differences to controls (**p* < 0.05, ***p* < 0.01, and ****p* < 0.001).

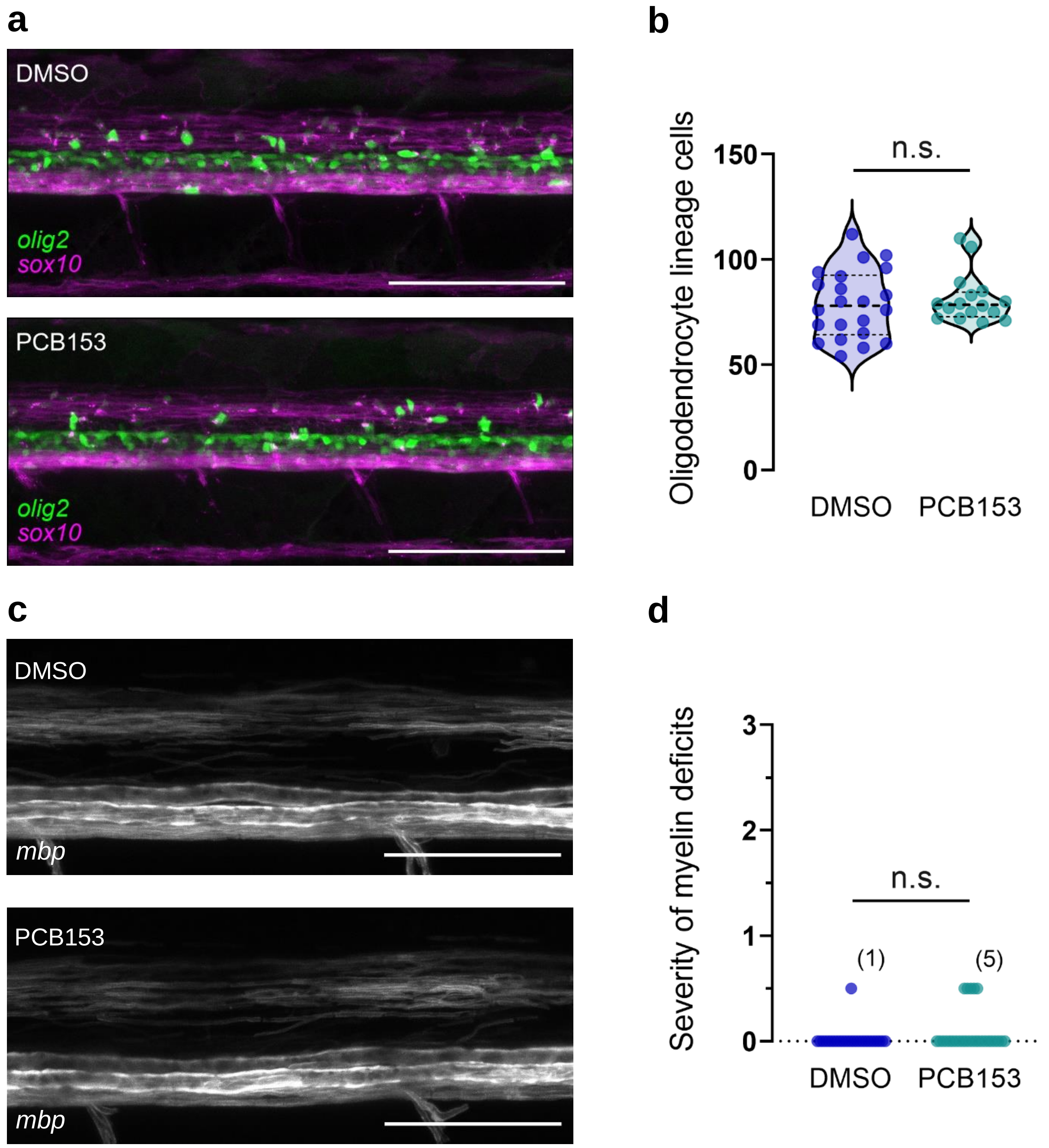

Supplementary Figure 7. Myelination in the spinal cord. (**a**) Representative images of oligodendrocyte precursor cells (OPC) and mature oligodendrocytes (green) in the spinal cords of DMSO (*n* = 22) and 1000 nM PCB153 (*n* = 16) treated *Tg(olig2:EGFP) x Tg(sox10:mRFP)* larvae at 6 dpf. Scale bar = 100 µm. (**b**) Quantification of oligodendrocyte lineage cells (green) per biological replicate within the same imaging area (319 µm x 160 µm) anterior of the spinal cord. Data points are biologically independent replicates from three independent experiments. Dashed lines represent median and quartiles. (**c**) Representative images of myelin sheath in the spinal cords of DMSO and 1000 nM PCB153 treated *Tg(mbp:EGFP-CAAX)* larvae at 6 dpf. Scale bar = 50 µm. (**d**) Classification of severity in myelin deficits in DMSO (*n* = 26) and 1000 nM PCB153 (*n* = 27) treated larvae according to Panlilio et al. 2020[1]. Data points are biologically independent replicates from three independent experiments.

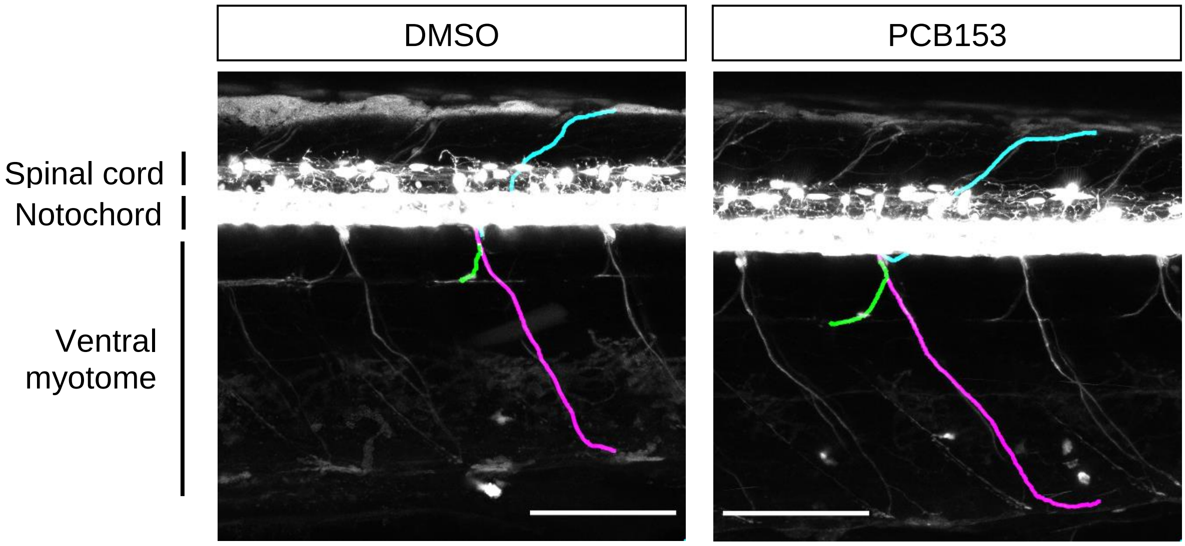

Supplementary Figure 8. Morphology of spinal cord primary motoneuron. Representative images of motoneuron morphology showing no apparent structural deficits in 1000 nM PCB153 exposed larvae (*n* = 18) in comparison to DMSO (*n* = 21). *Tg(olig2:EGFP)* zebrafish were imaged at 6 dpf. The different axons are color labeled as caudal primary (CaP, magenta), middle primary (MiP, cyan), and rostral primary (RoP, green) motoneuron. Scale bar 100 µm.

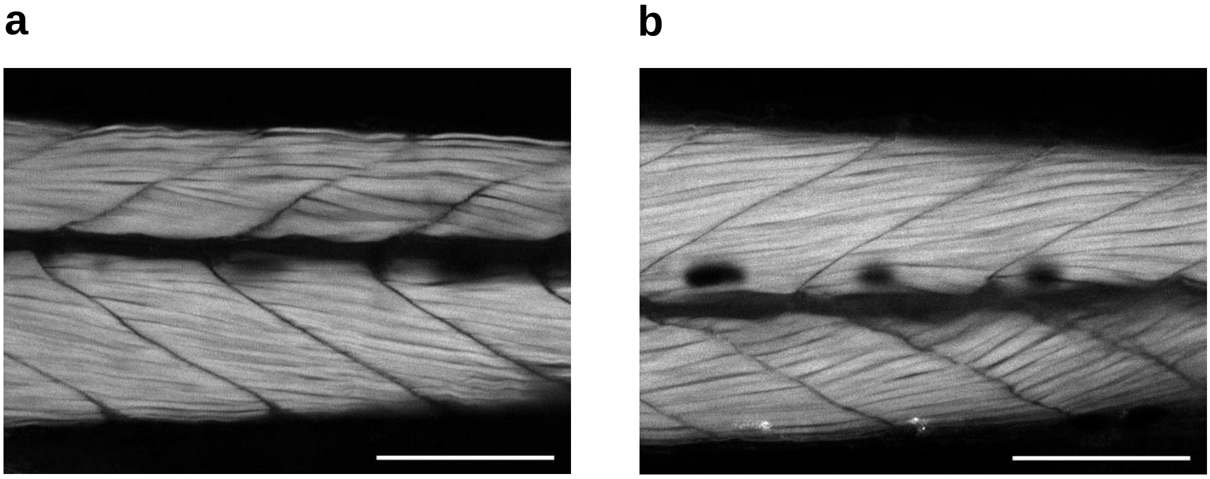

Supplementary Figure 9. Fast-twitch skeletal muscles. Representative images for (**a**) DMSO (*n* = 20) and (**b**) 1000 nM PCB153 (*n* = 20) treated larvae of trunk muscle fibers visualized using phalloidin, staining for polymerized actin. Images represent a slice of 1.2 µm thickness out of a z-stack. Scale bar: 100 µm.

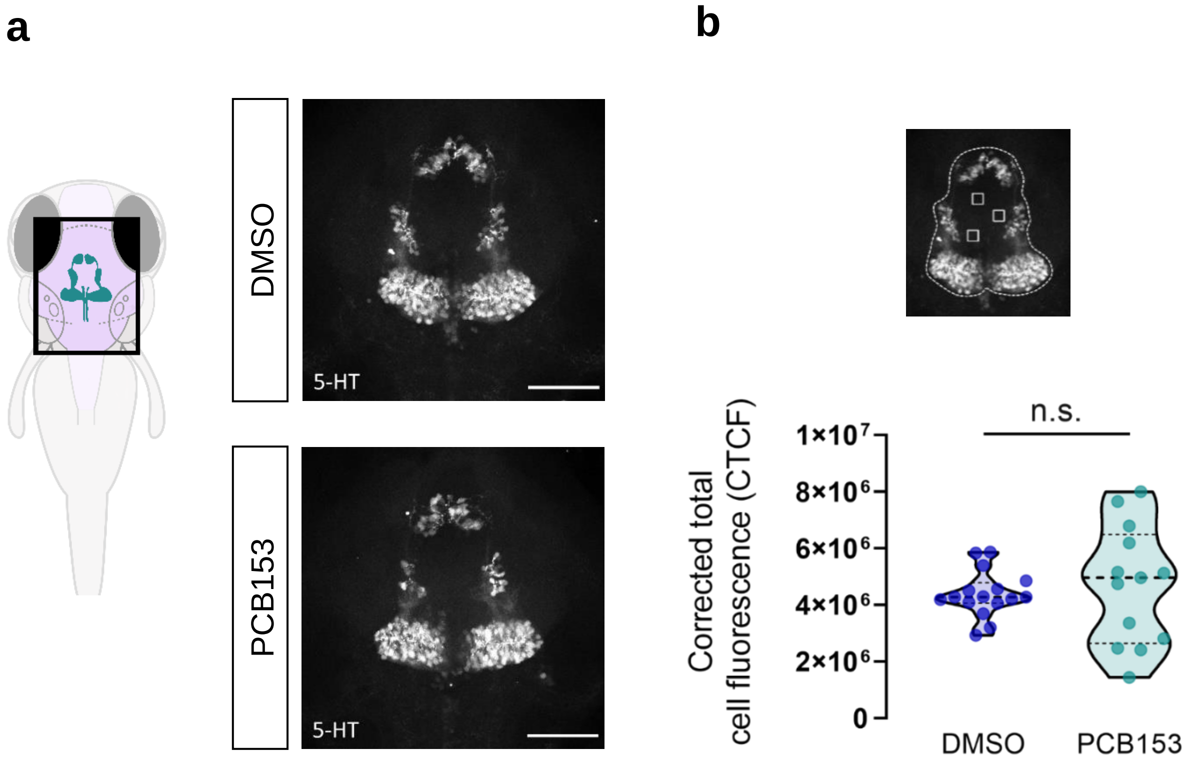

Supplementary Figure 10. Serotonin expression in the larval zebrafish brain. (**a**) Diagram indicating serotonergic neurons in the hypothalamus and representative images of serotonergic neurons in the hypothalamus of DMSO (*n* = 16) and 1000 nM PCB153 (*n* = 13) treated wild-type (AB) larvae at 6 dpf. Dissected brains were immunostained with anti-5-HT. Scale bar = 50 µm. (**b**) Quantification of 5-HT immunoreactive cell fluorescence calculated using the formula: Integrated density - (ROI x Mean fluorescence of background readings). The dashed line in the image denotes the ROI and the solid squares denote background readings. All data points are biologically independent replicates from three independent experiments. Dashed lines in the plot represent median and quartiles.

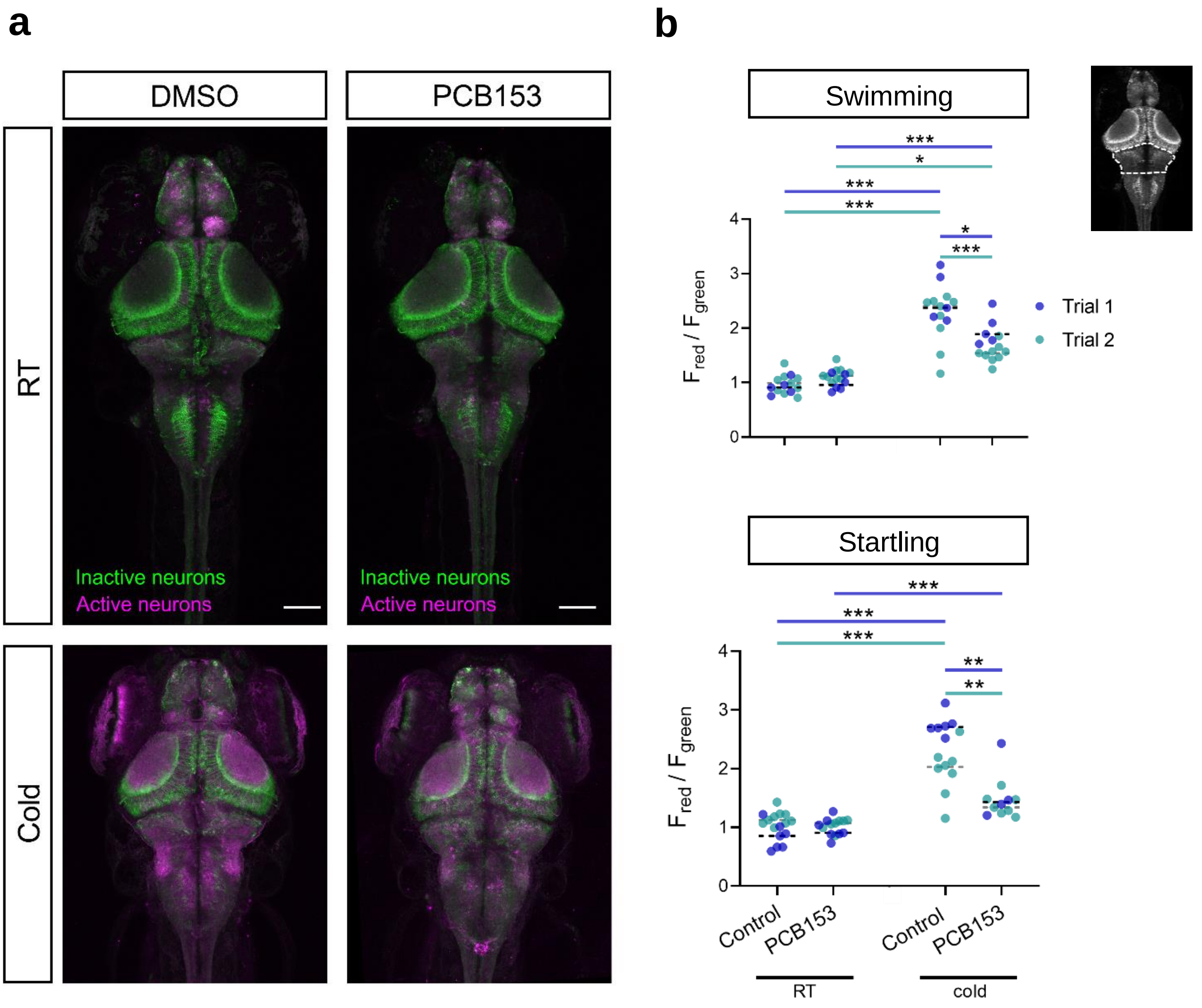

Supplementary Figure 11. Active neuronal populations in the brain. (**a**) Representative maximum intensity z-projection from a confocal stack of freely swimming 6 dpf DMSO (*n* = 15) and 1000 nM PCB153 (*n* = 15) exposed CaMPARI larvae at room temperature (RT) and at 4 °C (Cold). Active neurons had elevated calcium levels, which are photoconverted from green to red during UV exposure. Scale bar = 100 µm. (**b**) The ratio of red to green fluorescence intensity in the rostral hindbrain (indicated by a dashed line in image) in DMSO and 1000 nM PCB153 exposed freely swimming larvae and startling larvae at room temperature (RT) and in 4 ˚C (cold) medium. Asterisks indicate significant differences to controls (two-way ANOVA, *$p < 0.05$, **$p < 0.01$, and ***$p < 0.001$). Dashed lines in the plot represent the mean.

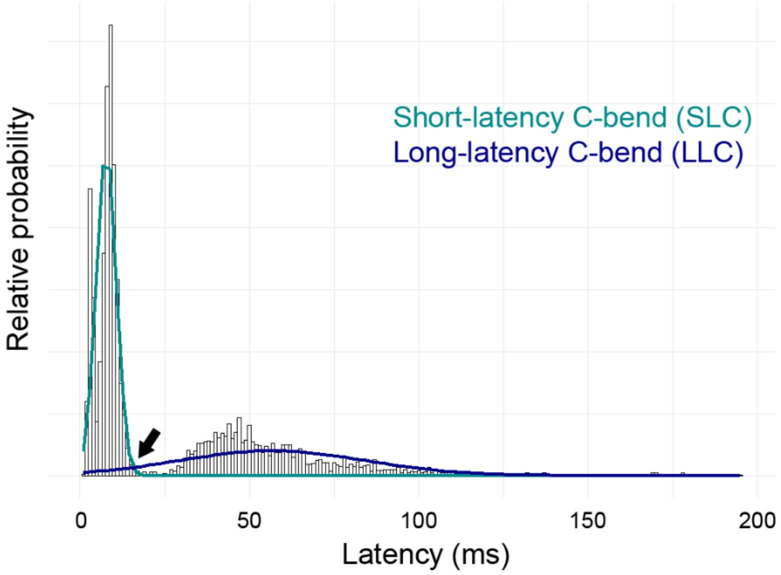

Supplementary Figure 12. Histogram of the latency distribution for control larvae. A two-component Gaussian mixture model was run to determine the cut-off by which there is a greater than 50% probability of a given data point belonging to either modeled distribution. The cut-off value is 15 milliseconds and is indicated by a solid arrow.

Supplementary Reference

1. Panlilio, J. M., Aluru, N. & Hahn, M. E. Developmental neurotoxicity of the harmful algal bloom toxin domoic acid: Cellular and molecular mechanisms underlying altered behavior in the zebrafish model. *Environ. Health Perspect.* **128**, 117002 (2020).
