# Supplementary material for: Developmental exposure to non-dioxin-like polychlorinated biphenyls promotes sensory deficits and disrupts dopaminergic and GABAergic signaling in zebrafish
Source: Commun Biol. 2021 Sep 24;4:1129. doi: 10.1038/s42003-021-02626-9 (PMC8463681; doi:10.1038/s42003-021-02626-9)
Supplement: Supplementary file 2 — Description of Supplementary Files [file 42003_2021_2626_MOESM2_ESM.pdf]

Description of Additional Supplementary Files

File name: Supplementary Movie 1

Description: Representative startle response of DMSO-treated (top two rows) and PCB153-treated (bottom two rows) zebrafish larvae at 6 dpf. Video was taken at 1000 frames s^{-1} for 250 ms with a vibrational stimulus (43 dB) after 13 frames.

File name: Supplementary Data 1

Description: Source data for graphs and charts.
