# Supplementary material for: Developmental exposure to non-dioxin-like polychlorinated biphenyls promotes sensory deficits and disrupts dopaminergic and GABAergic signaling in zebrafish
Source: Commun Biol. 2021 Sep 24;4:1129. doi: 10.1038/s42003-021-02626-9 (PMC8463681; doi:10.1038/s42003-021-02626-9)
Supplement: Supplementary file 5 — Reporting summary [file 42003_2021_2626_MOESM5_ESM.pdf]

nature research

Corresponding author(s): Nadja R Brun

Last updated by author(s): Jul 30, 2021

Reporting Summary

Nature Research wishes to improve the reproducibility of the work that we publish. This form provides structure for consistency and transparency in reporting. For further information on Nature Research policies, see our Editorial Policies and the Editorial Policy Checklist.

Statistics

For all statistical analyses, confirm that the following items are present in the figure legend, table legend, main text, or Methods section.

n/a	Confirmed	
☐	☒	The exact sample size (*n*) for each experimental group/condition, given as a discrete number and unit of measurement
☐	☒	A statement on whether measurements were taken from distinct samples or whether the same sample was measured repeatedly
☐	☒	The statistical test(s) used AND whether they are one- or two-sided<br>*Only common tests should be described solely by name; describe more complex techniques in the Methods section.*
☒	☐	A description of all covariates tested
☐	☒	A description of any assumptions or corrections, such as tests of normality and adjustment for multiple comparisons
☐	☒	A full description of the statistical parameters including central tendency (e.g. means) or other basic estimates (e.g. regression coefficient) AND variation (e.g. standard deviation) or associated estimates of uncertainty (e.g. confidence intervals)
☐	☒	For null hypothesis testing, the test statistic (e.g. *F*, *t*, *r*) with confidence intervals, effect sizes, degrees of freedom and *P* value noted<br>*Give P values as exact values whenever suitable.*
☒	☐	For Bayesian analysis, information on the choice of priors and Markov chain Monte Carlo settings
☒	☐	For hierarchical and complex designs, identification of the appropriate level for tests and full reporting of outcomes
☒	☐	Estimates of effect sizes (e.g. Cohen's *d*, Pearson's *r*), indicating how they were calculated

Our web collection on statistics for biologists contains articles on many of the points above.

Software and code

Policy information about availability of computer code

Data collection	Ethovision V12 (Noldus Information Technology)<br>Zeiss ZEN Blue (Zeiss Microscopy)<br>Custom Matlab code (Panlilio et al. 2020, Environmental Health Perspectives)
Data analysis	FLOTE software package (Burgess and Granato 2007, Journal of Neuroscience)<br>Ethovision V12 (Noldus Information Technology)<br>Fiji (Schindelin et al. 2012)<br>Custom Matlab code (Panlilio et al. 2020, Environmental Health Perspectives)<br>Custom R code (Panlilio et al. 2020, Environmental Health Perspectives)<br>PRISM V8.4 (GraphPad Software)

For manuscripts utilizing custom algorithms or software that are central to the research but not yet described in published literature, software must be made available to editors and reviewers. We strongly encourage code deposition in a community repository (e.g. GitHub). See the Nature Research guidelines for submitting code & software for further information.

Data

Policy information about availability of data

All manuscripts must include a data availability statement. This statement should provide the following information, where applicable:

- Accession codes, unique identifiers, or web links for publicly available datasets
- A list of figures that have associated raw data
- A description of any restrictions on data availability

The source data underlying the findings of this study are available within the Supplementary Data 1. Any remaining information and data such as images can be obtained from the corresponding author upon request.

Field-specific reporting

Please select the one below that is the best fit for your research. If you are not sure, read the appropriate sections before making your selection.

☒ Life sciences ☐ Behavioural & social sciences ☐ Ecological, evolutionary & environmental sciences

For a reference copy of the document with all sections, see nature.com/documents/nr-reporting-summary-flat.pdf

Life sciences study design

All studies must disclose on these points even when the disclosure is negative.

Sample size	Sample size was based on experience from previous published experiments leading to significant results
Data exclusions	No data were excluded.
Replication	All experiments were replicated at least three times except for the imaging with the CaMPARI zebrafish which was performed two times. All replications confirmed the findings.
Randomization	Zebrafish eggs for all exposures were always randomly picked and distributed into the vials or well-plates and after the distribution, the vials or well-plates were randomly labelled.
Blinding	Blinding during the experiment was not possible as the experiments were conducted by one scientist at a time and therefore the samples needed to be known and tracked. For image analysis, pictures were assigned numbers and the key was unknown to the evaluator.

Reporting for specific materials, systems and methods

We require information from authors about some types of materials, experimental systems and methods used in many studies. Here, indicate whether each material, system or method listed is relevant to your study. If you are not sure if a list item applies to your research, read the appropriate section before selecting a response.

Materials & experimental systems

n/a	Involved in the study	
☐	☒	Antibodies
☒	☐	Eukaryotic cell lines
☒	☐	Palaeontology and archaeology
☐	☒	Animals and other organisms
☒	☐	Human research participants
☒	☐	Clinical data
☒	☐	Dual use research of concern

Methods

n/a	Involved in the study	
☒	☐	ChIP-seq
☒	☐	Flow cytometry
☒	☐	MRI-based neuroimaging

Antibodies

Antibodies used	3A10 (Developmental Hybridoma Bank, antibody registry ID: AB 531874)<br>5-HT (Sigma-Aldrich, #S5545)<br>α-acetylated tubulin (Sigma-Aldrich, #T6793)
Validation	3A10<br>Axon guidance and the patterning of neuronal projections in vertebrates.<br>Jessell TM<br>Science (New York, N.Y.) 242.4879 (1988 Nov 4): 692-9.<br><br>5-HT

Transglutaminase Activity Determines Nuclear Localization of Serotonin Immunoreactivity in the Early Embryos of Invertebrates and Vertebrates.
Ivashkin E. et al.
ACS Chem Neurosci. 2019 Aug 21;10(8):3888-3899.

α-acetylated tubulin
Transglutaminase Activity Determines Nuclear Localization of Serotonin Immunoreactivity in the Early Embryos of Invertebrates and Vertebrates.
Ivashkin E. et al.
ACS Chem Neurosci. 2019 Aug 21;10(8):3888-3899.

Animals and other organisms

Policy information about studies involving animals; ARRIVE guidelines recommended for reporting animal research

Laboratory animals	Adult zebrafish lines used for obtaining eggs (males and females, 3-24 months old):<br>AB<br>Tg[elavl3:CaMPARI(W391F+V398L)]jf9<br>Tg(cntn1b:EGFP-CAAX)<br>Tg(olig2:EGFP)vu12<br>Tg(sox10:mRFP)<br>Tg(mbp:EGFP-CAAX)<br>Tg(mbp:EGFP)
Wild animals	The study did not involve wild animals.
Field-collected samples	The study did not involve samples collected from the field.
Ethics oversight	Zebrafish were handled in compliance with animal welfare regulations and maintained according to standard protocols (http://ZFIN.org). The culture and experimental procedures were approved by the Institutional Animal Care and Use Committee (ID Number BI21981.01).

Note that full information on the approval of the study protocol must also be provided in the manuscript.
